# From Diagnosis to Treatment: Navigating the Clinical Challenges of Dialyzer-Associated Thrombocytopenia

**DOI:** 10.7759/cureus.38891

**Published:** 2023-05-11

**Authors:** Ann Jose, Tony Varughese, Shreemayee De, Bisma Alam, Vishad Sheth

**Affiliations:** 1 Department of Internal Medicine, Hackensack University Medical Center, Hackensack, USA; 2 Department of Nephrology, Hackensack University Medical Center, Hackensack, USA; 3 Department of Pulmonary and Critical Care Medicine, Hackensack University Medical Center, Hackensack, USA

**Keywords:** hus, ttp, schistocytes, dialysis, thrombocytopenia

## Abstract

Thrombocytopenia is a common lab finding. The two fundamental groups are lack of production versus overconsumption of platelets. When common causes of thrombocytopenia have been ruled out and less common causes, such as thrombotic microangiopathic conditions, have been considered, it is important to keep in mind that patients undergoing dialysis may develop thrombocytopenia from the dialyzer itself.

This case is of a 51-year-old male who presented originally with celiac artery dissection and acute kidney injury requiring emergent dialysis. He ultimately developed thrombocytopenia during his hospitalization. It was initially presumed to be from thrombocytopenic purpura without improvement after plasmapheresis. No clear etiology was identified until it was suspected that the dialyzer was the source of thrombocytopenia. After changing the dialyzer type, the patient’s thrombocytopenia resolved.

Dialyzer-associated thrombocytopenia is a rare but reversible complication of hemodialysis. It is important to keep this differential in mind for patients undergoing hemodialysis.

## Introduction

Thrombocytopenia can be triggered by a variety of conditions. Some etiologies include drug-induced thrombocytopenia such as heparin-induced thrombocytopenia (HIT) and primary immune thrombocytopenic purpura; infections such as HIV or hepatitis C; and rheumatological conditions such as systemic lupus erythematosus, rheumatoid arthritis, and malignancy. Another major category includes thrombotic microangiopathy (TMA) such as thrombotic thrombocytopenic purpura (TTP) or hemolytic uremic syndrome (HUS) [1].

TMA such as TTP and HUS are among the first to be evaluated in cases of acute thrombocytopenia. One would confirm that the patient has microangiopathic hemolytic anemia (MAHA) as well as thrombocytopenia. The patient should undergo an evaluation for other systemic diseases which could lead to MAHA, and in the absence of these diseases, further evaluation needs to be conducted to determine the type of TMA [2]. History and physical examination, peripheral smear, and laboratory tests can help with an accurate diagnosis. Kidney biopsy contributes to the definitive diagnosis of TMA. Given its invasive nature, it is typically reserved for unclear cases. However, in the absence of TMA and unclear etiology of thrombocytopenia, iatrogenic causes must also be considered.

This is a case report of a 51-year-old male patient who had initially presented with celiac artery dissection and acute kidney injury requiring emergent hemodialysis. He then developed thrombocytopenia the day after hemodialysis was initiated. Extensive evaluation for the etiology of thrombocytopenia was conducted. However, it did not yield a definitive etiology. Upon realizing that the patient had developed thrombocytopenia after being started on hemodialysis, the possibility of dialyzer-associated thrombocytopenia was considered, with platelet levels improving upon switching to a different dialyzer.

## Case presentation

**Figure 1:**
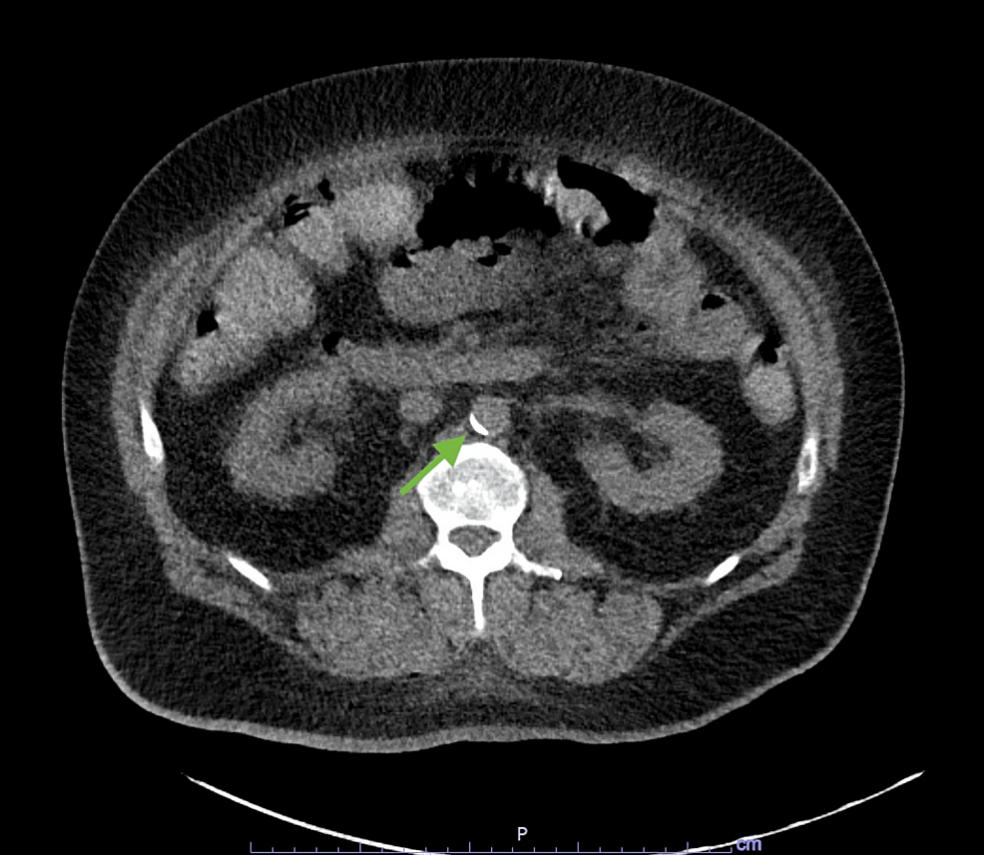
CT angiogram of the abdomen. Short segment occlusion of the origin of the celiac artery with distal reconstitution and associated periceliac infiltration.

A 51-year-old male with a past medical history of hypertension, dyslipidemia, and type 2 diabetes mellitus presented with severe back pain and dyspnea. A few weeks before his presentation, the patient and his wife had developed flu-like symptoms and attributed the symptoms to COVID-19. However, no confirmatory testing was done. Per the patient’s wife, he had been feeling progressively weaker and dyspneic and thus she brought him to the emergency department. As soon as the patient reached the emergency department, he started complaining of excruciating back pain. The patient was emergently intubated for acute hypoxic respiratory failure. CT angiogram showed occlusion of the celiac artery with distal reconstitution and surrounding infiltration (Figure 1). Findings were suspicious for celiac artery dissection. Vascular surgery was consulted with recommendations for non-surgical management with a heparin infusion to prevent reaccumulation of a clot.

**Figure 2:**
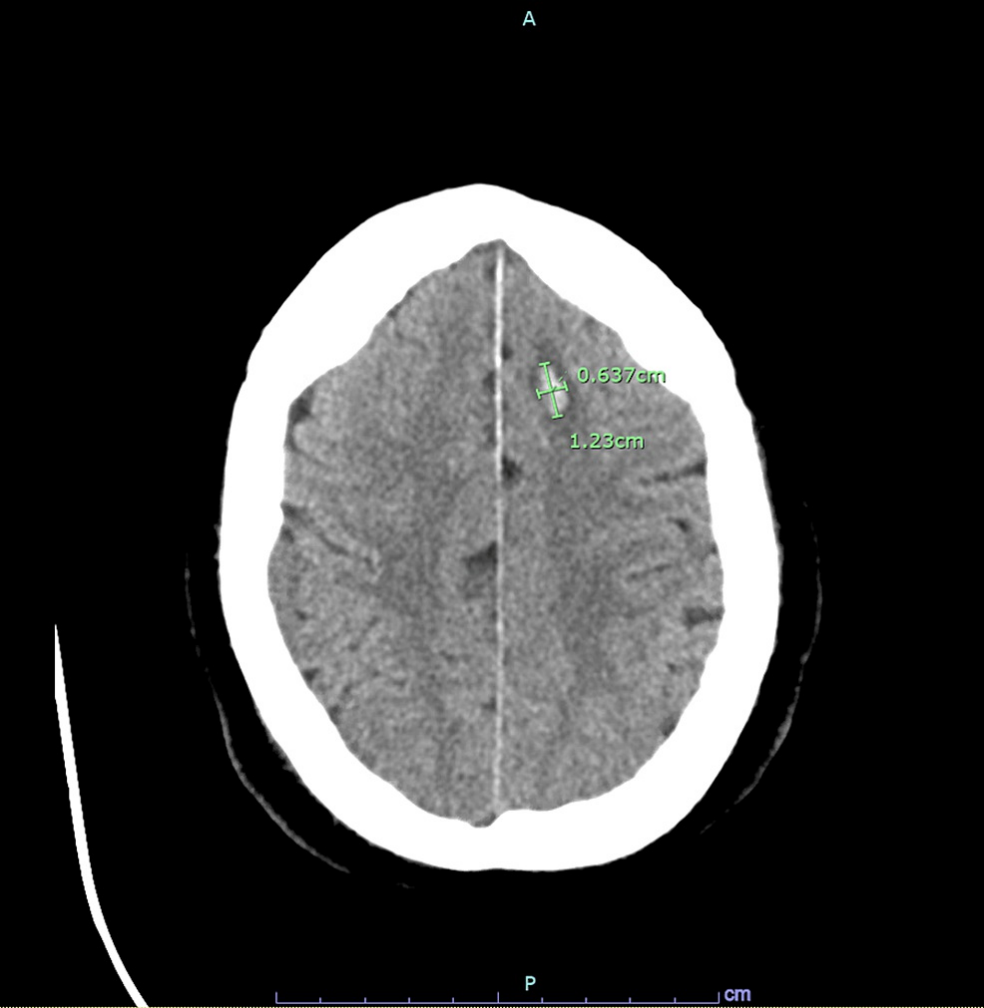
CT of the head without contrast. Small left frontal parenchymal hematoma measuring 1.23 × 0.637 cm in transaxial dimensions.

**Figure 3:**
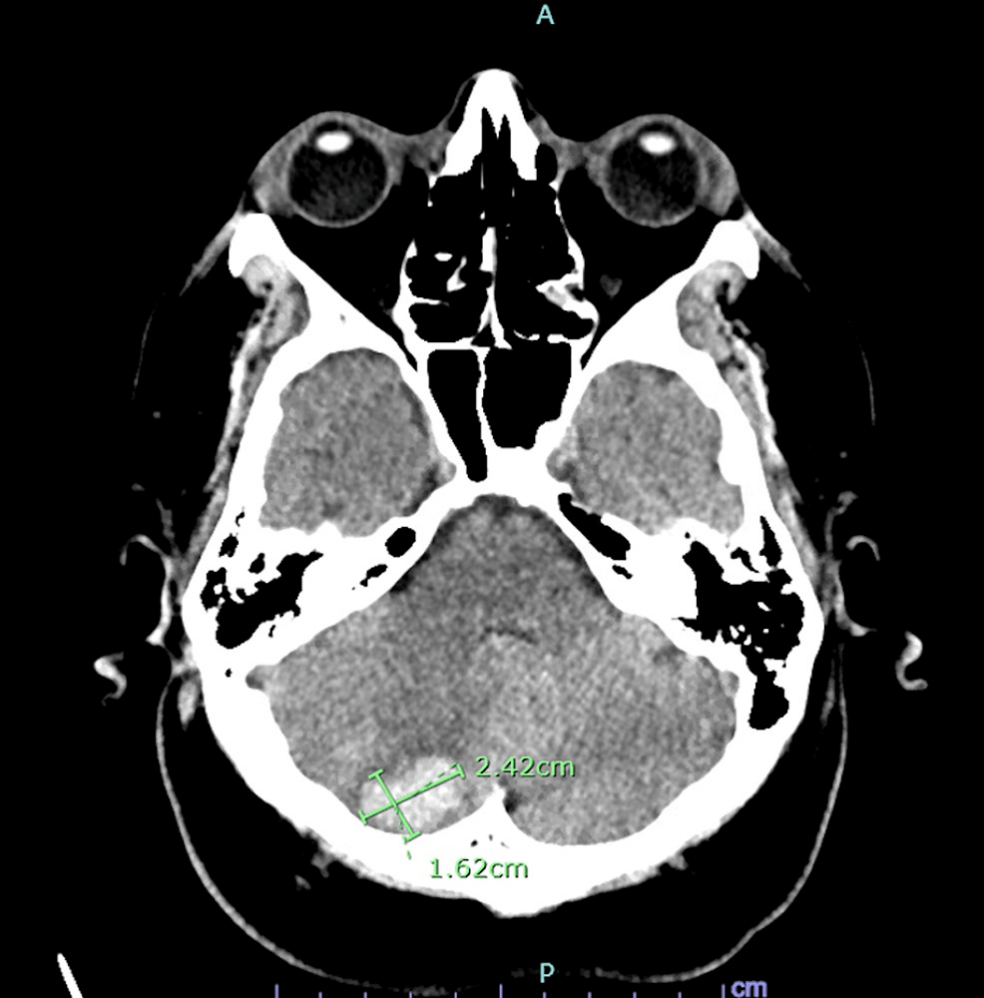
CT of the head without contrast. Moderate-sized right cerebellar parenchymal hematoma measuring 2.42 × 1.62 cm in transaxial dimensions. There is mild surrounding edema. Slight distortion of the adjacent fourth ventricle can be seen.

On admission, the patient had metabolic acidosis, acute kidney injury, and hyperkalemia (Table 1). He was emergently dialyzed. The patient’s platelets were 284 at the start of hemodialysis. Subsequent labs were significant for steeply decreasing platelet counts. Platelet count was 19 the day after hemodialysis was initiated and reached 8 two days later. During this time, there was a high suspicion of HIT, and thus, a heparin drip was discontinued and argatroban was initiated instead. The patient did not have any evidence of petechiae, purpura, or ecchymosis. He received multiple platelet transfusions for platelet levels below 10. Heparin-induced platelet antibody and serotonin release assay were within normal limits, and the ADAMSTS13 level was 1.0 (normal). He was weaned off sedation and was unable to follow commands. CT of the head showed two areas of intraparenchymal hemorrhage, and thus, argatroban was also stopped (Figures 2, 3). A repeat CT angiogram of the abdomen/pelvis revealed that the short segment proximal celiac axis total occlusion with distal reconstitution was improving from before and there was no evidence of bowel ischemia. Hematology was consulted due to a broad differential of TTP, HUS, disseminated intravascular coagulation (DIC), and vasculitis.

**Table 1:** Initial blood work. Patient’s blood work on presentation showing metabolic acidosis, acute kidney injury, and hyperkalemia. Platelet count is at a normal level.

Value	Reference range	Patient’s result
Hemoglobin	13–17 g/dL	12.0 g/dL
White blood cell	4–11 × 10^3^/µL	25.6 × 10^3^/µL
Platelet	135–430 × 10^3^/µL	313 × 10^3^/µL
Blood urea nitrogen	8–26 mg/dL	95 mg/dL
Creatinine	0.3–1.5 mg/dL	10.4 mg/dL
Sodium	136–145 mmol/L	137 mmol/L
Potassium	3.5–5.1 mmol/L	6.2 mmol/L
Bicarbonate	22–29 mmol/L	5 mmol/L
Lactic acid	0.5–2.0 mmol/L	18.7 mmol/L
pH	7.35–7.45	6.52
Prothrombin time	12.5–14.4 seconds	19.4 seconds
*Partial thromboplastin time*	25.1–34.4 seconds	34.7 seconds
International normalized ratio	0.9–1.10	1.60

Peripheral smear was significant for 5-10 schistocytes per high-powered field. On admission, lactate dehydrogenase was elevated at 1,714 U/L (downtrended to 586 U/L), haptoglobin was undetectable, and fibrinogen levels were normal. D-dimer was consistently elevated at >20 µg (0-0.5 µg). Plasmapheresis was initiated and the patient received four sessions with high-dose solumedrol 1 g for five days due to concern for TTP in addition to hemodialysis for volume overload and continued renal failure. A renal biopsy was performed, with findings significant for severe acute tubular necrosis but no findings of TMA. Chronic changes related to diabetic nephropathy and hypertension as well as some pigmented casts were seen. The patient also received one treatment of eculizumab for possible atypical HUS.

**Figure 4:**
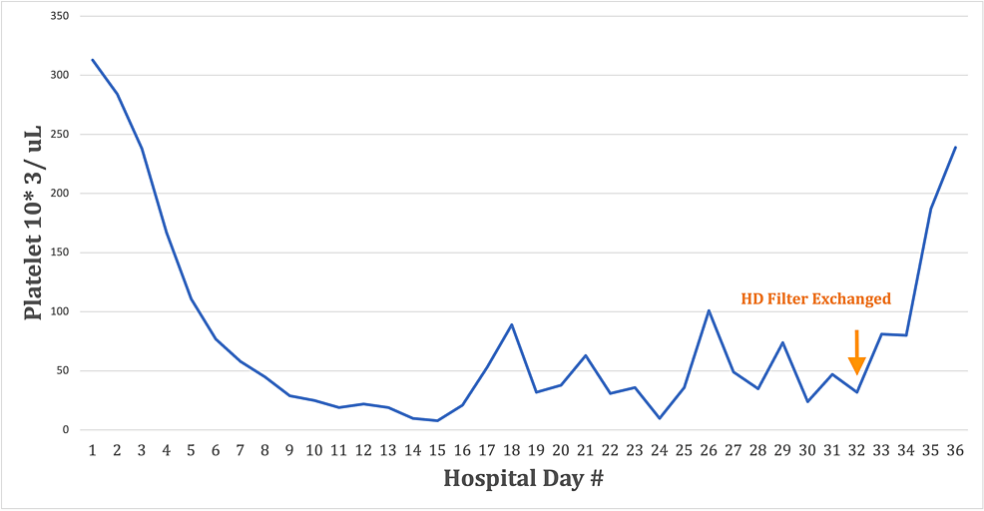
Daily platelet count in relation to hospital course. Platelet levels are plotted against hospital duration. Day 32 is delineated for when the hemodialysis filter was exchanged for Optiflux F160 Filter.

Platelet levels continued to fluctuate between 10 and 100, with multiple platelet transfusions to support levels. Exhaustive rheumatological and infectious workup was also conducted and was unremarkable. Further review revealed that thrombocytopenia had begun following the day hemodialysis was initiated. An iatrogenic cause of thrombocytopenia was considered, and the patient’s dialyzer was switched from Revaclear to Optiflux dialyzer. The platelet level was 32 on the day the dialyzer was exchanged. The following day the patient’s platelet level was 81, doubling to 187 the next day, then 239 the subsequent day, and was stable during the remaining duration of the patient’s hospitalization (Figure 4).

## Discussion

This case highlights the importance of making an accurate diagnosis of thrombocytopenia. Because our patient was initially treated with a heparin drip for celiac artery occlusion, there was moderate suspicion of HIT (4T score of 4). However, given the normal level of heparin-induced platelet antibody and serotonin release assay, HIT was excluded from the differential. The differential diagnoses included TTP, HUS, and DIC (International Society of Thrombosis and Hemostasis score of 6) as well. Thus, the patient was initiated on plasmapheresis and solumedrol for the potential treatment for TTP as the benefit of the intervention outweighed the risks. Typically with TTP, platelet transfusion is avoided. However, given the intraparenchymal brain hemorrhage noted on the CT head, the benefit of platelet transfusion appeared to outweigh the risks. The patient’s ADAMSTS13 inhibitor as well as von Willebrand factor protease activity level were found to be within normal range, thus excluding TTP. It was also noted that the patient’s platelet levels would respond appropriately after transfusion; however, they dropped shortly thereafter.

The patient’s peripheral smear was remarkable for 5-10 schistocytes per high-powered field. This can be seen with both HUS as well as DIC. The renal biopsy report showed only acute tubular necrosis and no TMA which is inconsistent with HUS. Factor V and VIII levels were also checked which did not follow the pattern of being consumed, as would be consistent with DIC. Dialysis-induced thrombocytopenia was considered a potential etiology. It should be noted that this is an extremely rare complication but an important differential diagnosis to keep in mind. Thrombocytopenia is observed in patients undergoing hemodialysis, but the underlying cause is important to explore. It is thought that reused polysulfone and cellulose acetate dialyzers can activate complement at cascade that ultimately causes an inflammatory response leading to thrombocytopenia [3,4].

A comprehensive workup for his thrombocytopenia throughout his hospitalization was unrevealing (Table 2). Due to the patient’s drop in platelets days into his hospitalization along with inconclusive laboratory findings, iatrogenic etiology as the cause of this thrombocytopenia was given further thought. Risk factors for dialysis-induced thrombocytopenia include age >70, autoimmune disease, primary kidney disease, lower platelet count baseline (109 × 10^3^/µL), and white blood cell change of post-dialysis of 6,175 cells/µL [5]. The only risk factor the patient had was leukocytic changes post-dialysis (which increased by 20,000 cells/µL after hemodialysis initially).

**Table 2:** Comprehensive thrombocytopenia blood work. Comprehensive thrombocytopenia workup for malignancy, infections, and autoimmune etiologies was unrevealing.

Value	Reference range	Patient’s result
Blood smear	-	5–10 and above schistocytes per high-power field, polychromasia, large platelets present, atypical lymphocytes, and toxic granulations of neutrophils
Flow cytometry	-	Flow cytometric analysis of the peripheral blood sample, after preferential gating of cells (CD45 bright, low side scatter gate - 9% of all events analyzed), highlights a predominant T-cell population without significant downregulation of the pan-T-cell antigens and a smaller population of polytypic B-cells. No definitive or distinct population of cells with aberrant characteristics and/or phenotypes is detected. No evidence of non-Hodgkin lymphoma
Fibrinogen	198–4,267 mg/dL	293
Factor II, V, VII, X	66%+	All within normal levels
Lactate dehydrogenase	125–220 U/L	3018
Haptoglobin	14–258 mg/dL	<8
Antineutrophilic cytoplasmic antibody (ANCA) screen	Evaluation for p-ANCA, MPO, PR3, c-ANCA, and atypical p-ANCA	Negative
*Epstein-Barr virus polymerase chain reaction*	-	Negative
Heparin-induced platelet antibodies, serotonin release assay	-	Negative
Anti-phospholipid syndrome (lupus anticoagulation, anti-cardiolipin, anti-β2-glycoprotein	-	Negative

The patient had improvement in his thrombocytopenia after switching to an Optiflux dialyzer. Optiflux dialyzers are composed of polysulfone. Ultimately, this composition lowers the activation of inflammatory factors that typically lead to complement activation [6]. As opposed to the patient’s original dialyzer (Revaclear) which has a Poracton membrane composed of polyarylethersulfone and polyvinylpyrrolidone blend. Synthetic membranes more commonly cause allergic reactions as opposed to cellular membranes, though polyarylethersulfone and polyvinylpyrrolidone blend more so than polysulfone alone [2]. The day before switching dialyzer the patient’s platelet levels were 32. On the subsequent days, the platelet levels were 81, 187, 239, and 262. The only change made was the dialyzer itself. It is important to be aware of this rare complication as the patient ultimately was being treated earlier with plasmapheresis, steroids, and platelet transfusions without success.

## Conclusions

Thrombocytopenia can be associated with a wide differential diagnosis. This case report demonstrates the importance of considering iatrogenic causes of thrombocytopenia. Dialyzer-associated thrombocytopenia is rare but should be considered in cases when hemodialysis is initiated and there is an acute drop in platelets, with unremarkable laboratory findings for other etiologies.
